# First fossil record of the mayfly family Vietnamellidae (Insecta, Ephemeroptera) from Burmese Amber confirms its Oriental origin and gives new insights into its evolution

**DOI:** 10.3897/zookeys.1036.66435

**Published:** 2021-05-10

**Authors:** Roman J. Godunko, Alexander V. Martynov, Arnold H. Staniczek

**Affiliations:** 1 Biology Centre of the Czech Academy of Sciences, Institute of Entomology, Branišovská 31, 37005 České Budějovice, Czech Republic Biology Centre of the Czech Academy of Sciences, Institute of Entomology České Budějovice Czech Republic; 2 Department of Invertebrate Zoology and Hydrobiology, University of Łódź, Banacha 12/16, 90237 Łódź, Poland University of Łódź Łódź Poland; 3 State Museum of Natural History, National Academy of Sciences of Ukraine, Teatralna 18, 79008, Lviv, Ukraine State Museum of Natural History, National Academy of Sciences of Ukraine Lviv Ukraine; 4 National Museum of Natural History, National Academy of Sciences of Ukraine, Bohdan Khmelnytsky 15, 01030, Kyiv, Ukraine National Museum of Natural History, National Academy of Sciences of Ukraine Kyiv Ukraine; 5 Department of Entomology, State Museum of Natural History Stuttgart, Rosenstein 1, 70191, Stuttgart, Germany State Museum of Natural History Stuttgart Stuttgart Germany

**Keywords:** *Burmella* gen. nov., Cretaceous, Ephemerelloidea, fossil mayflies, new genus, new species, Myanmar, Pannota

## Abstract

The small, monophyletic mayfly family Vietnamellidae Allen, 1984 has so far only been known from a few extant species of the genus *Vietnamella* Tshernova, 1972, which are all distributed in the Oriental Realm (Vietnam, Thailand, China, and India). Herein we report the first fossil record of Vietnamellidae based on a male and female imago from Mid-Cretaceous Burmese amber. We establish the new genus *Burmella***gen. nov.** to accommodate these two new Mesozoic specimens. Their attribution to Vietnamellidae is supported by the rounded shape of the hind wings with arched outer margin, the course of thoracic sutures, and characteristics of venation, especially of MP and Cu of the forewings and associated intercalary veins of the cubital field. At the same time, *Burmella***gen. nov.** clearly differs from *Vietnamella* by a diminished number of longitudinal and cross veins in the hind wings, and by the different shape of male genitalia. This first fossil record of Vietnamellidae supports an age of at least 100 Ma for this taxon.

## Introduction

The monogeneric family Vietnamellidae Allen, 1984 is generally regarded as monophyletic taxon within Pannota: Ephemerelloidea (Jacobus et al. 2005; Hu et al. 2017; Auychinda et al. 2020a). It was originally established by Tshernova (1972) with the type species *Vietnamella thani* Tshernova, 1972, based on larval specimens. The genus *Vietnamella* Tshernova, 1972 is endemic in the Oriental region with records from China, Thailand, India, and Vietnam (Tshernova 1972; Jacobus et al. 2005; Hu et al. 2017; Selvakumar et al. 2018; Auychinda et al. 2020a, b; Luo et al. 2020). So far there have been nine extant species formally described, which are *V. thani* Tshernova, 1972, *V. ornata* (Tshernova, 1972), *V. sinensis* (Hsu, 1936), *V. dabieshanensis* You & Su, 1987, *V. qingyuanensis* Zhou & Su, 1995, *V. guadunensis* Zhou & Su, 1995, *V. maculosa* Auychinda et al., 2020, *V. nanensis* Auchyinda et al., 2020, and *V. chebalingensis* Tong, 2020.

*Vietnamella dabieshanensis* You & Su, 1987, *V. qingyuanensis* Zhou & Su, 1995, and *V. guadunensis* Zhou & Su, 1995 are regarded as synonyms of *V. sinensis* (see Hu et al. 2017), which leaves at present six valid described species within *Vietnamella*. Additional records have been reported from India and Thailand, but these have not been identified to species level (Selvakumar et al. 2018; Auchyinda et al. 2020a, b), thus it is likely that there might be more extant species of *Vietnamella* discovered. However, so far there are no known fossil records of Vietnamellidae.

In this contribution, we present a fossil male and female adult mayfly specimen from Mesozoic Burmese Amber. These specimens are herein formally described as two new species in a new fossil genus *Burmella* gen. nov., which is placed within Vietnamellidae, thus constituting the first fossil record of this family.

## Materials and methods

The two specimens described in the present contribution are housed in the collection of the State Museum of Natural History Stuttgart (SMNS) under the inventory numbers BU-179 (holotype; male imago) and BU-321 (holotype; female imago). Both stones originate from the Hukawng Valley, Kachin State, Myanmar. The precise mine from which these stones originate is unknown. They were acquired from a local trader by Patrick Müller, Käshofen, Germany, who generously donated the amber pieces to the SMNS.

Hukawng amber was assigned to the Early Cretaceous, Upper Albian, with a maximum age of 98.79 ± 0.62 Ma, based on UePb zircon dating (see Shi et al. 2012), which is equivalent to the earliest Cenomanian (Gradstein et al. 2004). For more information on these amber deposits and their geological history see also Zherikhin and Ross (2000), Grimaldi et al. (2002), and Ross et al. (2010).

Drawings were made with a camera lucida on a Leica M205 C stereo microscope. Multiple photographs with different depth of field were taken through a Leica Z16 APO Macroscope equipped with a Leica DFC450 Digital Camera using Leica Application Suite v. 3.1.8. Photo stacks were processed with Helicon Focus Pro 6.4.1 to obtain combined photographs with extended depth of field, and subsequently enhanced with Adobe Photoshop CS3.

Anatomical terminology is based on Kluge (2004) and Bauernfeind and Soldán (2012).

## Systematic paleontology

### Subphylum Hexapoda Latreille, 1825

**Class Insecta Linnaeus, 1758**

**Order Ephemeroptera Hyatt & Arms, 1890**

**Family Vietnamellidae Allen, 1984**

#### Genus Burmella gen. nov.

Taxon classificationAnimaliaEphemeropteraVietnamellidae

3526B888-D327-5CC2-B5D1-DB7E34C24AB3

http://zoobank.org/0A29850B-F977-47E1-948E-37B5C617BD95

Figures 1 , 2 , 3 , 4 , 5 , 6 , 7 , 8 , 9 , 10 , Table 1

##### Type species.

*Burmella paucivenosa* sp. nov.

##### Derivation of name.

The generic name of female gender is a composition of “*Burmar*” as an ancient term for Myanmar, combined with “*ella*”, a common ending of generic names in mayflies, and especially so within Ephemerelloidea.

##### Diagnosis.

Adults of *Burmella* gen. nov. differ from other mayfly genera by the following combination of features: *forewings* (a) with small number of cross veins; (b) pterostigma with simple veins, not anastomosed; (c) CuP smoothly curved towards wing base; (d) two secondary bifurcate veins in cubital field; (e) at least several free marginal intercalaries along ventral margin; *hind wings* (f) strongly rounded, small, as long as 0.08–0.14 of forewing length; (g) small number of cross veins; (h) triad RS present or absent; no MA and MP triads; (i) no secondary branches of cubital veins; (j) costal process developed, rounded apically, situated centrally; *abdomen* (k) with vestigial gill sockets recognizable at least on segments II–VI; *genitalia* (l) with large median projection of styliger plate, widely rounded apically; (m) three distal segments of forceps strongly elongated and slender; segment II longest, 5× as long as segment III; segments III and IV approximately of equal length; segment IV expanding apically; (n) penis lobes widely separated by V-shaped cleft; (o) no trace of paracercus. Additionally, *in female* (p) anterior part of eyes covered by anterolaterally expanded clypeal shield.

Subimago and larva unknown.

##### Species composition.

*Burmella paucivenosa* sp. nov. (SMNS; BU-179); *Burmella clypeata* sp. nov. (SMNS; BU-321).

##### Locality and horizon.

Hukawng Valley, Kachin State, Myanmar (Burma); Cenomanian, mid-Cretaceous.

**Table 1.** Measurements of fossil representatives of the genus *Burmella* gen. nov.

Adult characters	*Burmella paucivenosa* sp. nov. [SMNS, BU-179, male imago] (mm)	*Burmella clypeata* sp. nov. [SMNS, BU-321, female imago] (mm)
Length of body	5.75	7.00
Length of right foreleg	2.51*	1.14*
Length of femur	0.83	0.42
Length of tibia	1.68	0.72*
Length of tarsus	–	–
Segment I	–	–
Segment II	–	–
Segment III	–	–
Segment IV	–	–
Segment V	–	–
Length of left foreleg	2.52*	1.64*
Length of femur	0.85	0.46
Length of tibia	1.67	1.18*
Length of tarsus	–	–
Segment I	–	–
Segment II	–	–
Segment III	–	–
Segment IV	–	–
Segment V	–	–
Length of right middle leg	2.78	–
Length of femur	1.45	–
Length of tibia	1.03	–
Length of tarsus	0.30	–
Segment I	0.08	–
Segment II	0.10	–
Segment III	0.10	–
Segment IV	0.11	–
Segment V	0.14	–
Length of left middle leg	2.70*	2.36
Length of femur	1.20	0.60
Length of tibia	1.02	1.34
Length of tarsus	0.48*	0.42
Segment I	–	0.08
Segment II	–	0.07
Segment III	–	0.07
Segment IV	–	0.08
Segment V	–	0.12
Length of right hind leg	2.47	1.71
Length of femur	1.02	0.71
Length of tibia	0.93	0.56
Length of tarsus	0.52	0.44
Segment I	0.07	0.09
Segment II	0.09	0.08
Segment III	0.10	0.07
Segment IV	0.12	0.08
Segment V	0.14	0.12
Length of left hind leg	2.07	2.60
Length of femur	1.00	0.98
Length of tibia	0.66	1.06
Length of tarsus	0.41	0.56
Segment I	0.05	0.11
Segment II	0.07	0.11
Segment III	0.07	0.10
Segment IV	0.10	0.10
Segment V	0.11	0.14
Length of right forewing	4.64	1.80
Length of left forewing	4.68	5.12
Length of right hind wing	0.66	0.45
Length of left hind wing	0.64	–
Hind/Fore wings length ratio	0.14	0.08
Length of cerci [right/left]	2.35*/1.24*	8.12/–

* preserved part.

#### Burmella paucivenosa sp. nov.

Taxon classificationAnimaliaEphemeropteraVietnamellidae

62645142-4D0B-5BE0-B0D9-47700EEC6F74

http://zoobank.org/FEE2D9CE-A89A-4C7E-8619-2CC736309DBC

**Figure 1.**
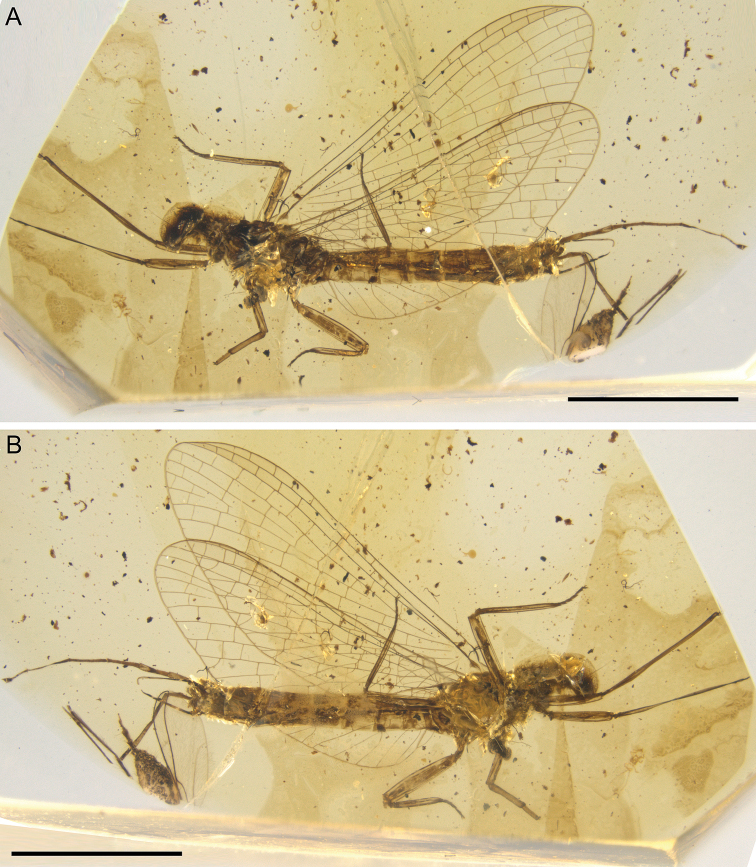
*Burmella paucivenosa* sp. nov., male imago, holotype, general lateral view **A** left side of body **B** right side of body. Scale bars: 2 mm.

**Figure 2.**
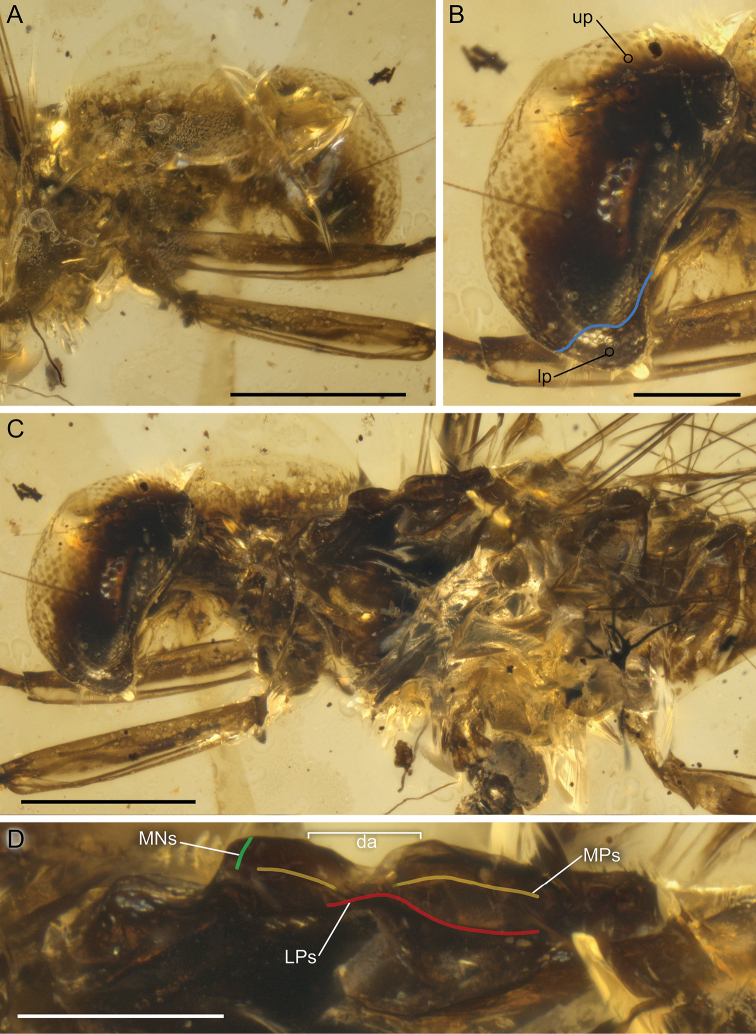
*Burmella paucivenosa* sp. nov., male imago, holotype **A** head, right lateral view **B** compound eye, left lateral view **C** head and thorax, left lateral view **D** mesothorax, left lateral view. Blue line – border between portions of compound eye, da – damaged area, lp – lower portion, LPs and red line – lateroparapsidal suture, MNs and green line – mesonotal suture, MPs and yellow line – medioparapsidal suture, up – upper portion. Scale bars: 0.5 mm (**A, C**); 0.2 mm (**B, D**).

**Figure 3.**
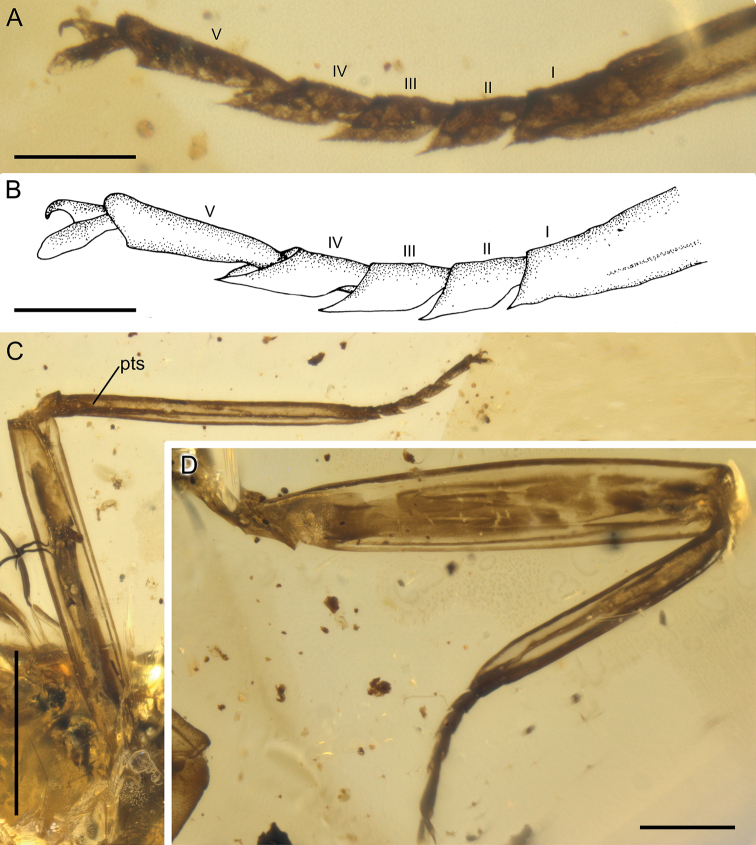
*Burmella paucivenosa* sp. nov., male imago, holotype **A, B** tarsus of right middle leg **C** right middle leg **D** left hind leg. I–V – tarsal segments, pts – patellotibial suture. Scale bars: 0.1 mm (**A, B**); 0.5 mm (**C**); 0.2 mm (**D**).

**Figure 4.**
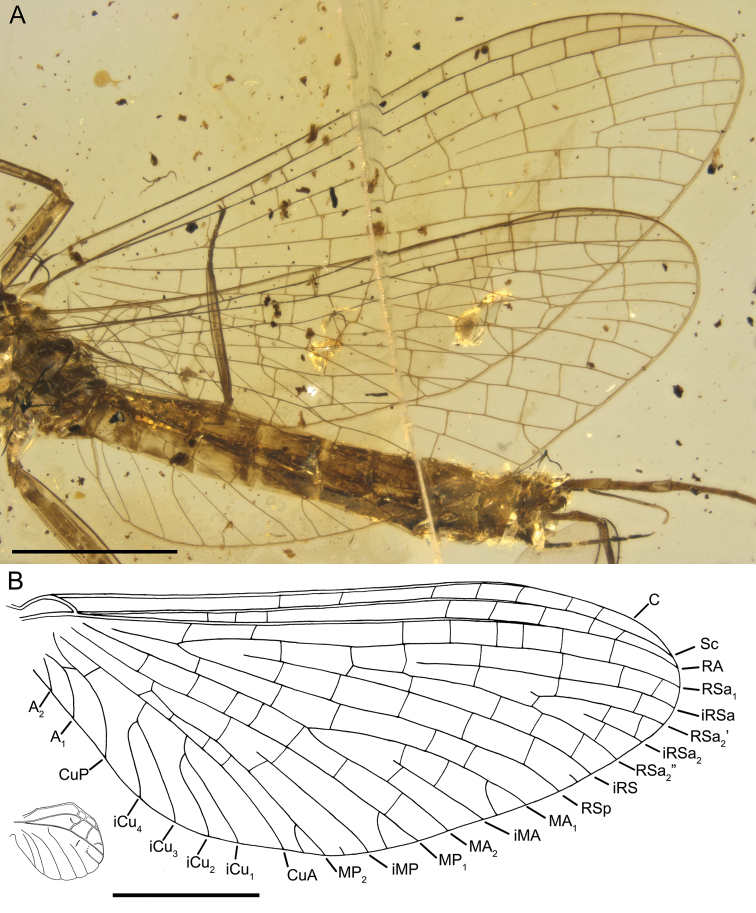
*Burmella paucivenosa* sp. nov., male imago, holotype **A** right and left wings position in amber **B** right forewing venation and size ratio of fore and hind wings. Scale bars: 1 mm.

**Figure 5.**
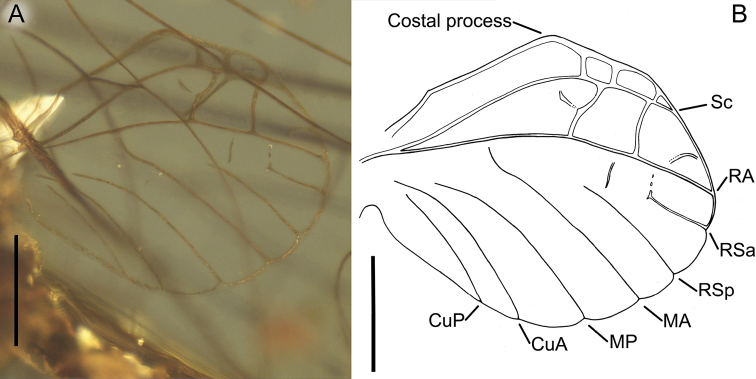
*Burmella paucivenosa* sp. nov., male imago, holotype **A** right hind wing in amber **B** right hind wing venation. Scale bars: 0.2 mm.

**Figure 6.**
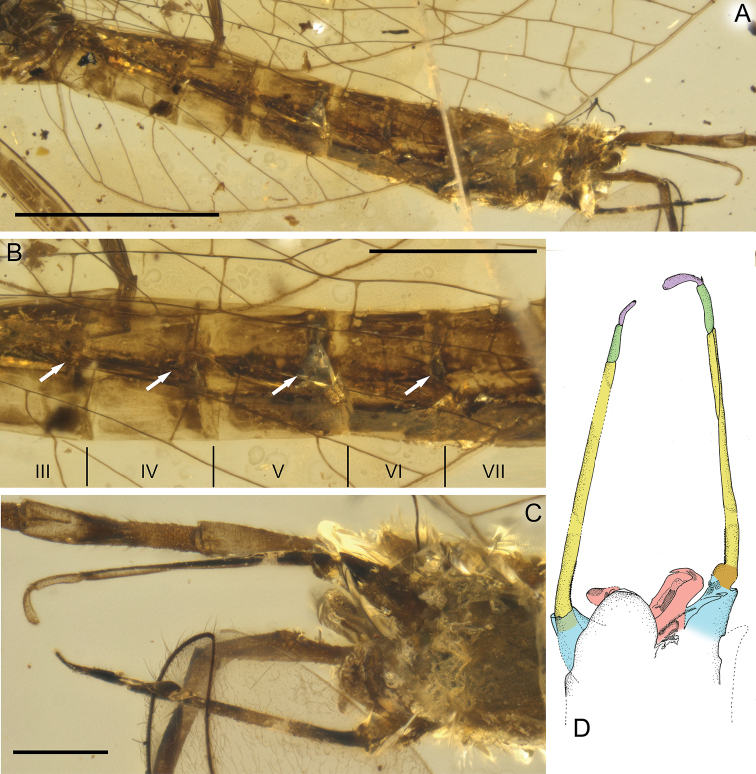
*Burmella paucivenosa* sp. nov., male imago, holotype **A** abdomen, left lateral view **B** abdominal segments III–VII, lateral view **C, D** genitalia, ventrolateral view. III–VII – numbers of segments, white arrows mark remnants of gill sockets, pink area – penis lobes, light blue area – gonobasis (styliger plate), brown area styliger segment I, yellow area –styliger segment II, light green area – styliger segment III, purple area – styliger segment IV. Scale bars: 1 mm (**A**); 0.5 mm (**B**); 0.2 mm (**C, D**).

Figures 1 , 2 , 3 , 4 , 5 , 6 , Table 1

##### Material examined.

***Holotype*.** Male imago in Mid-Cretaceous Burmese amber, SMNS collection, inventory number BU-179. Well preserved specimen visible in lateral aspect. Due to fragility, the piece of amber is additionally embedded in translucent resin to seal the specimen from oxygen and prevent mechanical damage. Body and both pairs of wings completely preserved (Figs 1, 4–5); most part of right and left foretibiae and both foretarsi missing; most part of caudal filaments missing. For measurements see Table 1.

##### Derivation of name.

The species epithet combines Latin “paucus”, few, and “venosus”, veined, referring to the reduced wing venation of the hind wing.

##### Diagnosis.

***Male imago*:** body length 5.75 mm; *forewings* with 3–4 marginal intercalaries connected with longitudinal veins, two free marginal intercalaries, no cross veins in anal field; *hind wings* strongly rounded, small, as long as 0.14× of forewing length, three cross veins between C–Sc, three cross veins between Sc–RA, one cross vein between RA–RSa, one cross vein between RA–RSp, RS not forked; *penis lobes* relatively simple, obliquely truncate apically, nearly tube-like; strong apical tooth on outer margin.

##### Description.

Colouration relatively pale, yellowish-brown to dark brown; eyes and mesonotum darkest, dark brown to blackish; abdominal segments partly translucent; traces of dark brown maculation along of lateral margins of terga (Figs 1, 2, 4, 6).

***Head*.** Compound eyes well-developed, large, widely rounded, medially contiguous; upper portion of compound eyes translucent and slightly yellowish apically, brownish-black basally; border between dorsal and ventral portions of compound eyes well distinguishable; lower portion of compound eyes brownish-black (Figs 1, 2A–C). Facets of compound eyes hexagonal. Ocelli poorly preserved, relatively small, without conspicuous colouration. Facial keel relatively small. Antennae slightly longer than head.

***Thorax*.** General colouration yellowish-brown to brownish-black. Prothorax narrow, light brown. Mesonotal suture transverse, distinctly expressed; medioparapsidal suture relatively straight; lateroparapsidal suture distinctly curved laterally; scutellum not modified; no preserved natural colouration of pigmented area of mesonotum. Mesosternum with brownish basisternum and slightly paler furcasternum; basisternum elongated; furcasternal protuberances distinctly separated. Lateral sides of mesothorax light brown to brown, with blackish maculation. Metathorax brown to dark brown, blackish maculation dorsally (Fig. 2C, D).

***Wings*.***Forewings* hyaline, translucent, relatively narrow; venation well recognizable, light brown to dark brown; veins darker proximally and slightly paler distally; relatively small number of cross veins, especially in medial, cubital, and anal fields; no jagged edge along of ventral margin of forewings. Pterostigma with 3–4 simple veins. Vein sections between C and RA slightly frosted-brown distally; veins C and Sc brown to dark brown, visible all over their length; RS forked near base, after 0.14 of its length; iRS well-developed, connected with RSp by 5 cross veins, not approximated to RSa_1_; MA fork slightly asymmetrical, forked after 0.60–0.62 of its length; MA_1_ and MA_2_ connected with iMA by 2–3 cross veins; MP asymmetrical, forked after 0.25 of its length, MP_1_ and MP_2_ basally connected by a single cross vein; iMP relatively short, connected with MP_1_ and MP_2_ by single cross veins from each side; CuP smoothly curved toward wing base, basally connected with CuA by cross vein cua–cup, CuP connected with A_1_ by cross vein cup-a_1_, cua–cup located distally from cup-a_1_; in cubital field two secondary bifurcate veins iCu_1+2_ and iCu_3+4_ arising from CuA (i.e. four veins iCu_1_–iCu_4_ each reaching basitornal margin of forewing); basal end of CuP closely approximated to CuA base; A_1_ closely approximated to A_2_; no cross veins in anal field. Several intercalaries (iRSa, iRSa_2_, iMA, iMP) connected to longitudinal veins by crossveins; two small, basally free marginal intercalaries in R and MP fields; no free intercalary veins in cubital and anal fields (Figs 1, 4).

***Hind wings*** hyaline, translucent, strongly rounded, small, as long as 0.14 of forewing length; venation light brown to brown; venation significantly simplified, with strong reduction of number of longitudinal and cross veins; ventral margin of hind wings without jagged edge. Few cross veins between C–Sc (3 veins), Sc–RA (3 veins), RA–RSa (one vein), and RA–RSp (one vein); no triads of RS, MA and MP; MA connected with R; MP approaching CuA; no secondary branches of cubital veins; no free marginal intercalaries; costal process rounded apically, markedly protruding above anterior wing margin, situated at nearly middle of hind wing length (Fig. 5A, B).

***Legs*** well preserved, except of tarsi missing in both forelegs; margins of preserved leg segments without visible strong spines or setae. For measurements of leg segments see Table 1.

***Right foreleg*:** length ratio of femur/tibia = 1/2.02; left foreleg: length ratio of femur/tibia = 1/1.96. Right middle leg completely preserved: length ratio of femur/tibia/tarsus = 1/0.71/0.21; length ratio of tarsomeres: 1/1.25/1.25/1.38/1.75 (5 > 4 > 3 = 2 > 1). Left middle leg much shorter than right one, probably re-grown after previous injury, therefore with changed proportions of tarsomeres. Right and left hind legs completely preserved; right hind leg: length ratio of femur/tibia/tarsus = 1/0.91/0.51; length ratio of tarsomeres: 1/1.29/1.43/1.71/2.00 (5 > 4 > 3 > 2 > 1). Left hind leg: length ratio of femur/tibia/tarsus = 1/0.66/0.41; length ratio of tarsomeres: 1/1.40/1.40/2.00/2.20 (5 > 4 > 3 = 2 > 1). Patellotibial suture present on middle and hind legs, absent on forelegs. First tarsomere of middle and hind legs fused with tibia. Claws ephemeropteroid on preserved middle and hind legs, with outer claw hooked and inner claw blunt (Figs 1A, B, 3A–D).

***Abdominal segments*** completely preserved, partly translucent, relatively pale, yellow to brown, with intensively brown maculation on terga laterally and sterna posteriorly. Vestigial gill sockets, not finger-like, recognizable on segments II–VI, poorly visible on segment VII due to influx of resin and cracks. Abdominal segments without large and prominent posterolateral projections; abdominal segments VIII–IX not elongated compared to previous segments. Abdominal sterna slightly paler than terga. Cerci brown, partly preserved; no trace of paracercus (Figs 1, 4, 6A, B).

***Genitalia*** well preserved, light brown to brown, darker maculation on forceps. Styliger plate angulate, mediocaudally deeply incised; median projection large, widely rounded apically, markedly protruding above anterior margin of styliger. Basal segment I of forceps short, with rounded inner margin, slightly wider than long; segment II of forceps strongly elongated, slender distal segments III and IV much shorter, approximately of equal length; segment IV expanding apically; length ratio of forceps segments II–IV: 1.00/0.20/0.18 (Fig. 6C, D). Penis lobes widely separated by V-shaped cleft, relatively simple, obliquely truncate apically, nearly tube-like; structure of left penis lobe poorly visible; inner side of right penis lobe probably partly damaged or lost (i.e. looks semicircular from ventral side); strong apical tooth on outer margin; titillators not distinguishable (Fig. 6C, D).

##### Affinities.

*Burmella paucivenosa* sp. nov. exhibits a combination of morphological characters allowing its attribution to Vietnamellidae, namely the presence of strongly rounded hind wings in combination with the presence of short intercalaries distally connected with longitudinal veins. Compared to other representatives of Vietnamellidae, *Burmella paucivenosa* sp. nov. is characterized by the presence of only two short free marginal intercalaries, while the number of these intercalary veins in all extant species and also in *Burmella clypeata* sp. nov. is significantly higher.

Within Vietnamellidae, *Burmella paucivenosa* sp. nov. can be attributed to the newly described genus *Burmella* gen. nov., as defined in Diagnosis (see above), mainly based on the following diagnostic characters: shape and structure of venation of hind wings, with reduced cross venation and distinct costal process situated centrally; lack of furcation of RS, MA, MP, CuA, and CuP in hind wings (Fig. 5); shape of male genitalia with deeply diverted penis lobes (Fig. 6C, D).

In the latter character, the male imago of *Burmella paucivenosa* sp. nov. differs from all other known male adults of Vietnamellidae. The genus *Vietnamella* is characterized by the presence of a club-shaped, elongated penis that is medially fused along its longitudinal axis, with only a small, V- or U-shaped incision apically (Tshernova 1972: 613, fig. 7; Hu et al. 2017: 385, figs 4C, 5C; Auychinda et al. 2020a: 8, fig. 4G; 2020b: 28, figs 7J, K, 8J, K). In contrast to *Vietnamella*, the tubular penis of *Burmella paucivenosa* sp. nov. is medially deeply split, with lobes strongly stretched laterally (Fig. 6C, D). Obvious differences are also visible in shape and proportions of forceps segments. In *Burmella paucivenosa* sp. nov. the 4-segmented forceps is strongly elongated and slender, with segment II being the longest, with the same width distally as segment III basally, while distal segment IV is markedly elongated and nearly subequal to segment III (Fig. 6C, D). On the contrary, in *Vietnamella* the forceps is only 3-segmented with segments significantly different in shape and proportions: Segment I is the longest one, while shortest segment III is small and rounded, which is typical for many species of Ephemerelloidea (see Kluge 2004).

#### Burmella clypeata sp. nov.

Taxon classificationAnimaliaEphemeropteraVietnamellidae

2B8AFB00-5E55-53E7-B37A-3633A025E34E

http://zoobank.org/5F823D68-5C03-4138-8875-D80917D61452

**Figure 7.**
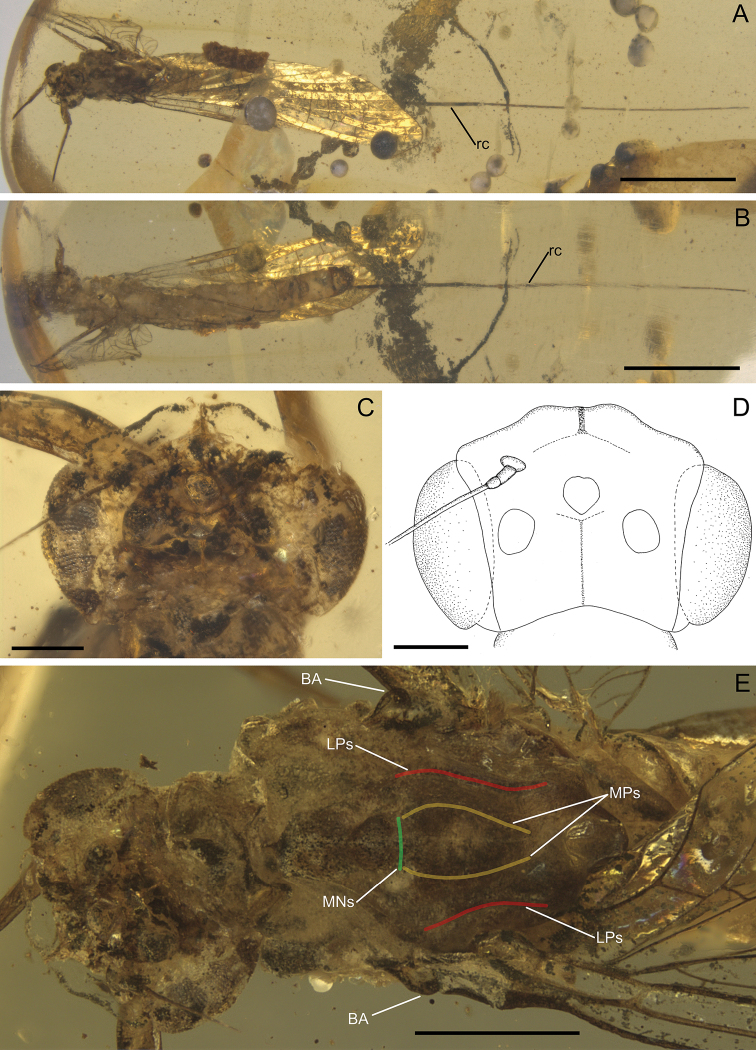
*Burmella clypeata* sp. nov., female imago, holotype **A** general dorsal view **B** general ventral view **C, D** head, dorsal view **E** head and thorax, dorsal view. BA – basal sclerite (basalare), LPs and red line – lateroparapsidal suture, MNs and green line – mesonotal suture, MPs and yellow line – medioparapsidal suture, rc – right cercus. Scale bars: 2 mm (**A, B**); 0.2 mm (**C, D**); 0.5 mm (**E**).

**Figure 8.**
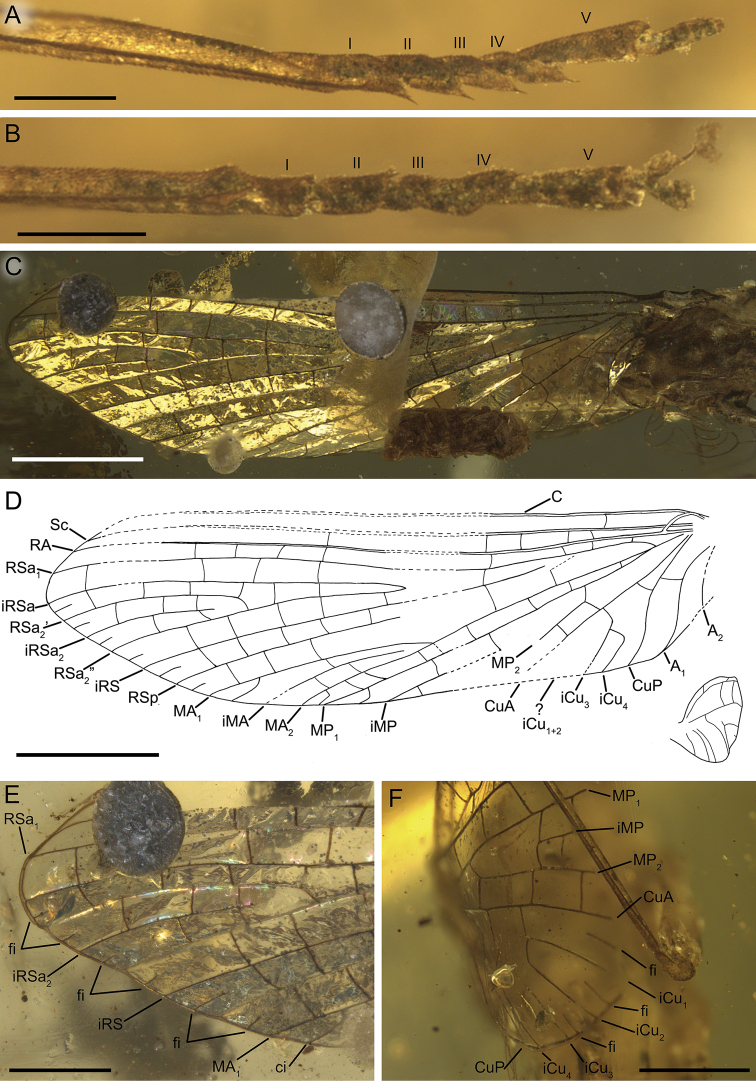
*Burmella clypeata* sp. nov., female imago, holotype **A** tarsus of left hind leg **B** tarsus of right hind leg **C** left forewing in amber **D** left forewing venation and size ratio of left fore- and right hind wings **E** distal part of left forewing **F** preserved basal part of right forewing. I–V – tarsal segments, ci – basally connected intercalary vein, fi – basally free intercalary vein. Scale bars: 0.1 mm (**A, B**); 1 mm (**C, D**); 0.5 mm (**E, F**).

**Figure 9.**
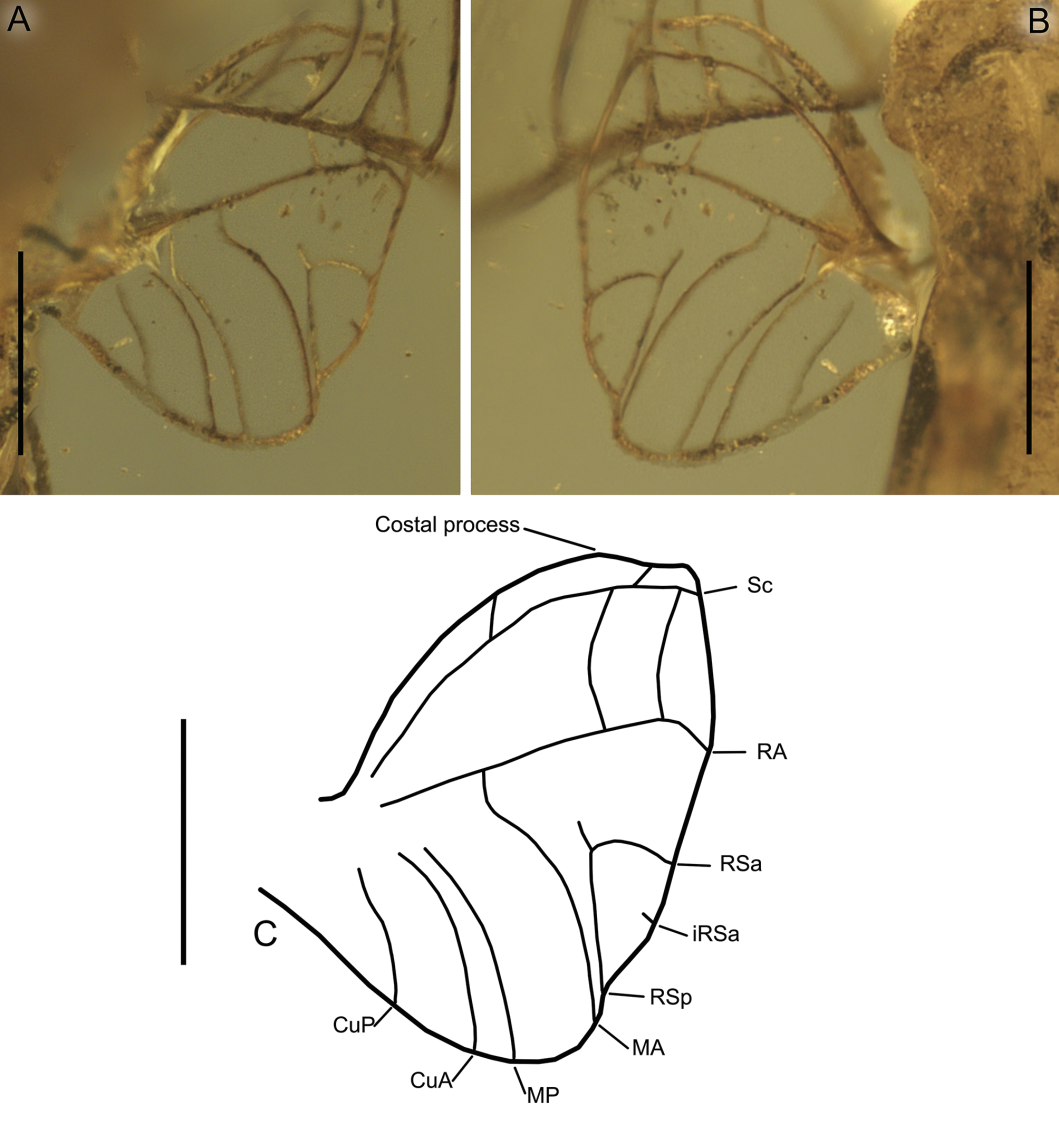
*Burmella clypeata* sp. nov., female imago, holotype **A** right hind wing, dorsal view **B** right hind wing, ventral view **C** right hind wing venation. Scale bars: 0.2 mm (**A, B**); 0.5 mm (**C**).

**Figure 10.**
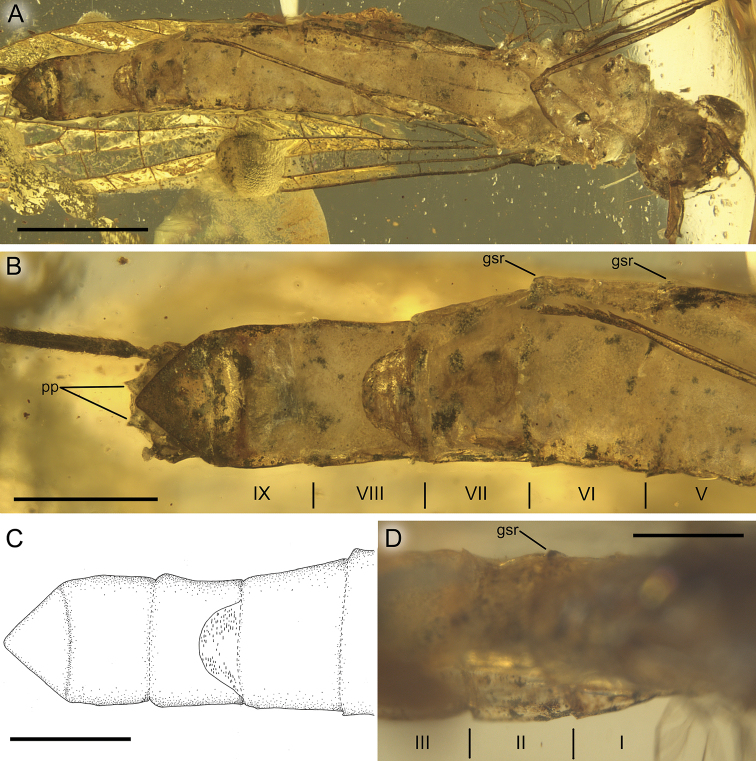
*Burmella clypeata* sp. nov., female imago, holotype **A** body, ventral view **B, C** apical part of abdomen, ventral view **D** basal segments of abdomen, dorsal view. I–III and V–IX – numbers of abdominal segments, gsr – remnant of gill socket, pp – paraproct plate. Scale bars: 1 mm (**A**); 0.5 mm (**B–D**).

Figures 7 , 8 , 9 , 10 , Table 1

##### Material examined.

***Holotype*.** Female imago in Mid-Cretaceous Burmese amber, SMNS collection, inventory number: BU-321. Well preserved specimen visible in dorsal/ventral aspect. Body and left forewings preserved except of lost distal part of C and Sc; left forewing twisted, covering dorsal side of abdomen; right forewings twisted, only partly preserved, distal part missing; foretibiae damaged; right antenna, foretarsi, right middle leg and left cercus missing (Figs 7, 8, 10). Left hind wing not visible. For measurements see Table 1.

##### Derivation of name.

The species epithet refers to the laterally expanded clypeus that partly covers the eyes.

##### Diagnosis.

***Female imago*:** body length 7.00 mm; *forewings* with at least four short marginal intercalaries in MA–MP field basally attached to longitudinal veins, six free marginal intercalaries in RS field; *hind wing* strongly rounded, small, as long as 0.08× of forewing length, two cross veins between C–Sc, two cross veins between Sc–RA; RS forked; *subgenital plate* more than 2.00× as wide as long, convex and widely rounded apically; *subanal plate* triangular, elongated, rounded apically without cleft.

##### Description.

General colouration of body relatively pale, light brown to dark brown. Ventral side of body slightly darker than dorsal side. Body covered by blackish maculation (Figs 7A, B, 10).

***Head*.** Clypeus expanded anterolaterally, partly covering anterior part of eyes. Eyes brown, elongated, relatively large, widely separated medially; facets of eyes hexagonal. Distance between eyes 0.73× of head width. Ocelli well preserved, large, without conspicuous colouration. Facial keel small. Antenna brown, approximately as long as head; segmentation hardly distinguishable, therefore not depicted (see Fig. 7C–D).

***Thorax*.** General colouration brown to dark brown. Lateral aspect of thorax not visible. Prothorax narrow, brown. Mesonotal suture transverse, expressed; medioparapsidal suture poorly visible, straight; lateroparapsidal suture distinctly curved laterally; no preserved natural colouration of pigmented area of mesonotum. Ventral side of mesothorax poorly visible; basisternum relatively short and wide distally, furcasternal protuberances distinctly separated. Metathorax brown to dark brown, blackish maculation dorsally (Fig. 7E).

***Wings*.***Forewings* hyaline, translucent, relatively narrow; venation poorly recognizable due to wing deformation, pollution on surface and resin influxes [left wing], and damage of distal part [right wing]; venation well visible from dorsal, and partly from lateral side. Veins light brown to brown; relatively small number of cross veins; no jagged edge along of ventral margin (Fig. 8C–F).

General pattern of forewing venation similar to those of male imago of *Burmella paucivenosa* sp. nov., except for the following features: six free intercalary veins at least in RS field and CuA–CuP; at least four intercalaries in MA–MP field basally attached to longitudinal veins (Fig. 8C–F).

***Hind wings*** hyaline, translucent, small, as long as 0.08 of forewing length; preserved wing is deformed due to embedding, but most probable was naturally strongly rounded, with shallow costal process; venation brown, significantly simplifies; strong reduction of number of longitudinal and cross veins; no jagged edge along of ventral margin. General structure and pattern of hind wing venation similar to those in male imago of *Burmella paucivenosa* sp. nov., except for the following features: a few cross veins between C–Sc (2 veins), and Sc–RA (2 veins); fork RS present, iRS short, no cross veins in RS field; costal process not prominent (Fig. 9A–C).

***Legs*** well preserved, except for both forelegs with partly missing tibiae and tarsi; no visible strong spines or setae on margins of leg segments. Preserved part of forelegs darker than middle and hind legs, brown to intensively brown (Fig. 8A, B). For measurements of leg segments see Table 1.

Forelegs partly preserved [due to damage of foretibiae the ratio of femur/tibia is not calculated]. Left middle leg completely preserved: length ratio of femur/tibia/tarsus = 1/2.23/0.70; length ratio of tarsomeres: 1/0.88/0.88/1.00/1.50 (5 > 4 > 3 = 2 < 1). Right hind leg much shorter than left one, probably re-grown after previous injury, therefore with changed proportions of tarsomeres: length ratio of femur/tibia/tarsus =1/0.79/0.62; length ratio of tarsomeres: 1/0.89/0.78/0.89/1.33 (5 > 4 > 3 < 2 < 1). Left hind leg: length ratio of femur/tibia/tarsus = 1/1.02/0.57; length ratio of tarsomeres: 1/1/0.91/0.91/1.27 (5 > 4 = 3 < 2 = 1) (Figs 8A, B, 10A). Other leg characters similar to those in male imago of *Burmella paucivenosa* sp. nov.

***Abdominal segments*** completely preserved, light brown to brown, with blackish maculation on terga and sterna; ventral side of abdomen paler than dorsal side. Vestigial gill sockets, not finger-like, well recognizable on segments II, V, and IV; on other segments gill sockets not distinguishable due to body position in amber. Abdominal segments without large and prominent posterolateral projections; no conspicuous elongation of distal segments compared to proximal ones. Subgenital plate relatively broad, more than 2.00× as wide as long, convex and widely rounded apically. Subanal plate triangular, elongated, moderately narrow and rounded apically without apical cleft. Right cercus completely preserved, brown, darker proximally, approximately as long as body (Fig. 10A–D).

##### Affinities.

Attribution of *Burmella clypeata* sp. nov. to the newly described genus is confirmed based on the shape of hind wings, and specific venation.

On the other hand, some aspects of the venation of fore- and hind wings differ between *Burmella clypeata* sp. nov. and *Burmella paucivenosa* sp. nov. The forewings of *Burmella clypeata* sp. nov. differ by the presence of numerous free marginal intercalaries between iRS and CuP, as well as the presence of at least one cross vein between A_1_ and A_2_. In the hind wings differences between the extinct species described here refer to the number of cross veins between C–Sc and Sc–RA. The presence of RS furcation and blunt costal process in *Burmella clypeata* sp. nov. are also suitable for the separation of both species. In contrast to all other representatives of Vietnamellidae, the clypeus in the female of *Burmella clypeata* sp. nov. is anterolaterally expanded, as a result the anterior portion of eyes is partly covered by this clypeal shield (Fig. 7C–D; compare with e.g., Auychinda et al. 2020a: 9, figs A–E; 2020b: 30, fig. 9A).

We do however not per se exclude a possible conspecifity of both fossil specimens. This may be supported by a similar, small body size of both specimens, with similar proportions of male/female body length as in extant Vietnamellidae (for *Burmella* gen. nov. the ratio is 0.82; for *Vietnamella* between 0.92 and 0.96). Also, the anterolaterally expanded clypeus in *B. clypeata* may not exclude their conspecifity. Similar clypeal expansions present in one sex only have been reported in several extant and fossil species of Heptageniidae (e.g. in the subgenus Ecdyonurus (Nestormeus) Godunko, 2004), representing a morphological trait independently occurring in several unrelated taxa within the family (see Godunko 2007: 66, figs 1, 2; Hrivniak et al. 2018: 199, 204–205, figs 2–5). However, a clear difference in the venation of fore- and hind wings between *B. paucivenosa* sp. nov. and *B. clypeata* sp. nov. rather points to the presence of two different fossil species.

In any case, unless specimens of different sex are syninclusions and fossilized in mating position, we tend to describe males and females of the same genus as different species also to maintain nomenclatural stability (see e.g. Staniczek and Godunko 2016; Godunko et al. 2019).

### Remarks on the systematic position of *Burmella* gen. nov. as genus within Vietnamellidae (Ephemerelloidea)

Based on the available evidence, we propose the systematic position of *Burmella* gen. nov. as a distinct congener of the family Vietnamellidae, although its character distribution implies disturbing homoplasy of some characters within Ephemerelloidea (see also McCafferty and Wang 2000; Kluge 2004; Ogden et al. 2009).

However, *Burmella* gen. nov. shares most important apomorphic characters of Ephemerelloidea (compare also Kluge 2004):

(1) The basal connection of CuP with CuA and A 1 by associated cross veins cua–cup and cup-a 1. As typical for most Ephemerelloidea, cua–cup in Burmella gen. nov. is located more distally than cup-a 1;
(2) The specific arrangement of the cubital field with one or more bifurcated veins is another apomorphic feature of Ephemerelloidea, which is also present in Burmella gen. nov.;
(3) The apomorphic arrangement of thoracic sutures in Ephemerelloidea is also present in Burmella gen. nov., namely a transverse mesonotal suture and a lateroparap sidal suture with laterally curved posterior end, which is associated with specific lateral sclerotized area;
(4) The separated furcasternal protuberances count as yet another apomorphic character supporting this placement.

Within Ephemerelloidea, *Burmella* gen. nov. shares the main wing apomorphy of Vietnamellidae, which is presence of strongly rounded hind wings with moderately arched foremargin. Except of Vietnamellidae, such a rounded shape of hind wings is only known in extant (*Baetisca* Walsh, 1863) and fossil (*Balticobaetisca* Staniczek & Bechly, 2002) genera of the family Baetiscidae Edmunds & Traver, 1954 (Staniczek and Bechly 2002: 8; Kluge 2004: 67, fig. 17C; Godunko and Krzemiński 2009: 127, fig. 1). However, the anteritornous forewing shape of *Burmella* gen. nov. as well as all the aforementioned apomorphic characters of Ephemerelloidea precludes closer affinities with the posteritornous Baetiscidae. At the same time, the presence of marginal intercalaries attached to longitudinal veins, and the presence of free intercalary veins of forewings, also fits well with the character distribution in Ephemerelloidea.

While the overall character distribution accounts for a placement of *Burmella* gen. nov. within Vietnamellidae, there are also considerable differences between the herein described species and the extant genus *Vietnamella* that justify its placement in a separate genus:

(1) A smaller number of cross veins in forewings, with only few, simple cross veins in pterostigma (in contrast to a well-developed cross venation, with simple and forked veins in the pterostigmatic area of Vietnamella) (Figs 1, 4, 8D–F; compare with Auychinda et al. 2020a: 28–30, figs 7H, 8H, 9H; 2020b: 9, fig. 5F);
(2) CuP of forewings smoothly curved toward the hind margin of wing and basally directed toward CuA (in contrast to CuP of Vietnamella, which is sharply curved at approximately 1/3 of its length) (Figs 4B, 8D; compare e.g. Kluge 2004: 318, fig. 95A);
(3) Distinctly diminished hind wings, as long as 0.08–0.14 of forewing length, with a few cross veins only (in contrast to Vietnamella, with well-developed cross venation and fore/hind length ratio at least 0.20) (Figs 5, 9; compare with Kluge 2004: 318, fig. 95B; Auychinda et al. 2020a: 28–30, figs 7I, 8I, 9I; 2020b, 9, fig. 5G);
(4) Cubital field of hind wings without secondary branches and cross venation, with simple CuA and CuP only (in contrast to well-developed branches of CuA and CuP in Vietnamella, connected by several cross veins) (Figs 5, 9);
(5) Rounded apically costal process of hind wings situated centrally (in Vietnamella costal process of hind wings is absent, with leading margin slightly concave centrally) (Figs 5, 9);
(6) Only a single longitudinal intercalary vein [iMP] between MP 1 and MP 2 of forewings (in contrast to Vietnamella, with iMP and 2–5 additional elongated intercalaries between MP 1 and MP 2) (Figs 1, 4, 8D; compare with Kluge 2004: 318, fig. 95A; Auychinda et al. 2020a: 28–30, figs 7H, 8H, 9H; 2020b, 9, fig. 5F);
(7) There are no traces of pacacercus present (in Vietnamella a well-developed paracercus is present) (Figs 1, 4A, 6A, 6C, 10A, B; compare with Auychinda et al. 2020a: 31, fig. 10C Auychinda et al. 2020b, fig. 5N);
(8) Both fossil specimens are significantly smaller than known adults of Vietnamella. While the body size of male and female adults of Vietnamella varies within 12–17 mm, the adults of Burmella gen. nov. are approximately 2× smaller (5.75 mm the male, and 7.00 mm the female). The ratio of body length of fossil male and female is 0.82, which is a little less in compare to Vietnamella (the ratio is 0.92–0.96).

At the same time, *Burmella* gen. nov. shows significant differences in the structure and shape of male genitalia compared to both *Vietnamella* and other Ephemerelloidea. While the 3-segmented forceps of *Vietnamella* is relatively short, with enlarged segment I, a well recognizable border between segments I and II, and a nearly rounded, small, distal segment, the 4-segmented forceps of *Burmella* gen. nov. are even more elongated and slender, with two short segments distally. The longest segment II is distally as wide as the base of the segment III, and elongated segment IV is expanding apically. The penis lobes of *Burmella* gen. nov. are deeply separated by a V-shaped cleft and outstretched laterally. Overall, the genital morphology and arrangement of forceps segments appears to be a rather plesiomorphic condition resembling conditions like in Siphlonuridae Ulmer, 1920 or Heptageniidae Needham, 1901.

This plesiomorphic condition of the genitalia however would imply a convergent, parallel development of both the 3-segmented forceps with elongated first segment and only one short segment distally, and a medially fused, stab-like penis in both *Vietnamella* and other Ephemerelloidea (Kluge 2004). However, all other synapomorphies *Burmella* gen. nov. shares with *Vietnamella* and other Ephemerelloidea in our opinion clearly outweigh the genital characters and justify its placement within Vietnamellidae. A more thorough phylogenetic discussion after a cladistic analysis will be conducted as soon as new Mesozoic material becomes available (Staniczek et al. in prep.)

The discovery of *Burmella* gen. nov. in about 100 Ma old Burmese amber however points to a surprisingly old age of Vietnamellidae, at the same time indicating that the major splits of Ephemerelloidea might have occurred earlier than previously assumed (Staniczek et al. 2018). Its discovery makes an Oriental origin of the group likely and supports the assumption of Vietnamellidae as endemic Oriental group within Ephemerelloidea.

## Supplementary Material

XML Treatment for Burmella

XML Treatment for Burmella paucivenosa

XML Treatment for Burmella clypeata
